# Dysfunctional Lipid‐Induced Secretion of Glucagon‐Like Peptide‐2(GLP‐2), but Not of Incretins Glucagon‐Like Peptide‐1(GLP‐1)/Glucose‐Dependent Insulinotropic Polypeptide(GIP), Promotes Metabolic Dysfunction–Associated Steatotic Liver Disease (MASLD) Onset and Progression Through Gut Barrier Disruption and Endotoxemia

**DOI:** 10.1002/mco2.70323

**Published:** 2026-04-02

**Authors:** Giovanni Musso, Silvia Pinach, Alberto Mella, Franco De Michieli, Anna Calabrese, Deborah Trifiro, Giovanna Petrangolini, Maurizio Cassader, Filippo Mariano, Roberto Gambino

**Affiliations:** ^1^ MECAU Department San Luigi Gonzaga Hospital, Orbassano Turin Italy; ^2^ Department of Medical Sciences Città Della Salute e della Scienza Hospital University of Turin Torino Italy; ^3^ Department of Nephrology Città Della Salute e della Scienza Hospital University of Turin Torino Italy; ^4^ R & D Indena S.p.A. Milan Italy

**Keywords:** endotoxemia, intestinal barrier, LPS, NF‐kB, postprandial, steatohepatitis

1

Metabolic dysfunction–associated steatotic liver disease (MASLD) encompasses a histological spectrum ranging from simple steatosis (Metabolic dysfunction–associated steatotic liver, MASL) to progressive metabolic dysfunction–associated steatohepatitis (MASH) [1], the latter being the most rapidly growing indication for liver transplantation [1].

Diverse genetic, nutritional, hormonal, and inflammatory factors have been implicated in lipotoxicity, which is the pathogenic hallmark of MASLD, but their roles in MASLD onset and progression from steatosis to MASH are unclear.

Among nutritional factors, dietary fat has been linked to obesity and type 2 diabetes (T2DM) in the general population, and to liver disease progression in experimental MASH models, while data in humans are conflicting [2]. Our modern eating pattern of several meals during the day places us in a postprandial state for most of the day, and the role of impaired postprandial homeostasis in the development of T2DM and atherosclerosis is increasingly recognized [3]: consistently, recent guidelines endorse nonfasting plasma lipids’ measurement to better assess cardiovascular risk [4].

The consequences of fat ingestion depend on the interplay between the nature of ingested fat and individual metabolic plasticity: even in healthy lean, insulin sensitive individuals, a single oral saturated fat meal transiently increases hepatic triglyceride content by 35%, upregulates upstream pathways of hepatic inflammation, including the key proinflammatory and profibrotic transcription factor nuclear factor (NF)‐kB, and decreases insulin sensitivity for up to 6 h after the meal [5]. In healthy individuals, such detrimental consequences are rapidly reversed by a dynamic array of postprandial hormonal responses from the gut, the liver, and adipose tissue, including incretins GLP‐1 and GIP, gut‐derived intestinotrophic peptide GLP‐2, hepatokine fibroblast growth factor (FGF)‐21, and adipokines, which limit fat‐induced inflammation and lipotoxicity [6]. The role of each of these mechanisms in MASLD onset and progression remains undefined, as human MASLD studies typically focus on static, fasting conditions. Hypothesizing that a maladaptive response to fat ingestion promotes MASLD onset and progression, we investigated:

1. dynamic response to fat ingestion of gut‐derived entero‐endocrine peptides and incretins, intestinal barrier, endotoxemia, hepatokines, and adipokines in patients with biopsy‐proven MASL, MASH, and matched controls without liver disease;
2. the effect of a pharmacological intervention with phospholipid curcumin, a natural lipophilic polyphenol and a known stimulator of secretion of glucagon‐like peptides via translational and post‐translational mechanisms [7, 8], on these adaptive responses and on liver disease in MASH.

We analyzed two independent cohorts: a cross‐sectional cohort and an interventional (longitudinal) cohort of individuals.

The cross‐sectional cohort was represented by 104 biopsy‐proven MASLD patients (52 with MASL and 52 with MASH) and 52 controls without liver disease enrolled at two medical centers (HUMANITAS Gradenigo, Turin, Italy, and Città della Salute e della Scienza, Turin, Italy), between January 22, 2020 and July 24, 2022.

MASLD was diagnosed and staged according to current guidelines [1] and hepatic steatosis was ruled out in controls as detailed in the Supporting Information.

MASH, MASL, and controls were matched for age, gender, BMI category (normal weight/overweight/obese), abdominal obesity (present/absent, as assessed by waist circumference), diabetes status(present/absent), and a number of metabolic syndrome criteria (Table S1) to limit the confounding effect of these conditions on postprandial entero‐hormonal response.

Use of GLP‐1 receptor agonists, dipeptidy peptidase (DPP) IV inhibitors and Sodium‐Glucose Transport Protein 2 (SGLT2) Inhibitors at any time during the study was an exclusion criterion.

The interventional cohort represented by 52 biopsy‐proven MASH was enrolled in a 72‐week double‐blind placebo‐controlled randomized trial with phospholipid curcumin Meriva 2 g/d, as previously reported [9]. Meriva and placebo groups were also matched for age, gender, obesity category, diabetes status, and a number of metabolic syndrome criteria [9].

All participants underwent a standardized oral fat tolerance test (OFTT) within 2 months of clinical, biochemical, and histological (for MASLD patients) assessment (Supporting Information).

MASH patients enrolled in the RCT repeated all baseline assessments after 72 weeks at end‐of‐treatment (EOT), including liver biopsy and OFTT.

Dietary and physical activity record and genetic analyses were made as described in the Supporting Information.

All participants underwent a standardized 8‐h OFTT [8]. The following blood parameters were measured:

1. plasma total cholesterol, Tg, HDL‐C, apolipoprotein A1 (ApoA1), apolipoprotein B48 (apoB48), NEFAs; oxLDLs, glucose, and insulin**;**
2. entero‐endocrine peptides: incretins (GLP‐1 and GIP) and intestinotrophic peptide GLP‐2;
3. intestinal barrier integrity markers: circulating levels of the tight junction protein Zonulin;
4. endotoxemia: bacterial lipopolysaccharide (LPS);
5. hepatokine: FGF‐21;
6. adipokines adiponectin, resistin;
7. NF‐kB activation in circulating mononuclear cells (MNCs) (detailed in the Supporting Information);
8. chemokines Monocyte Chemoattractant Protein (MCP)‐1;
9. markers of hepatocyte apoptosis. Serum caspase‐cleaved cytokeratin‐18 (CK‐18 M30) CK‐18 M30 (M30 Apoptosense ELISA Kit; PEVIVA AB, Bromma, Sweden);
10. markers of active fibrogenesis: lumican.

All analytic methods are detailed in the Supporting Information.

The activation of the pro‐inflammatory transcription factor NF‐κB in liver cells in MASLD was assessed by liver tissue immunohistochemistry (IHC) as detailed in the Supporting Information.

Sample size calculation and statistical analyses are detailed in the Supporting Information.

The main results of the study were as follows.

In the cross‐sectional cohort, the postprandial GLP‐2 response was progressively impaired, while postprandial Zonulin response, endotoxemia, and NF‐kB activation progressively increased across controls, MASL, and MASH groups, despite similar fasting values across the three groups (Table S2, Figure 1 left panels).

**FIGURE 1:**
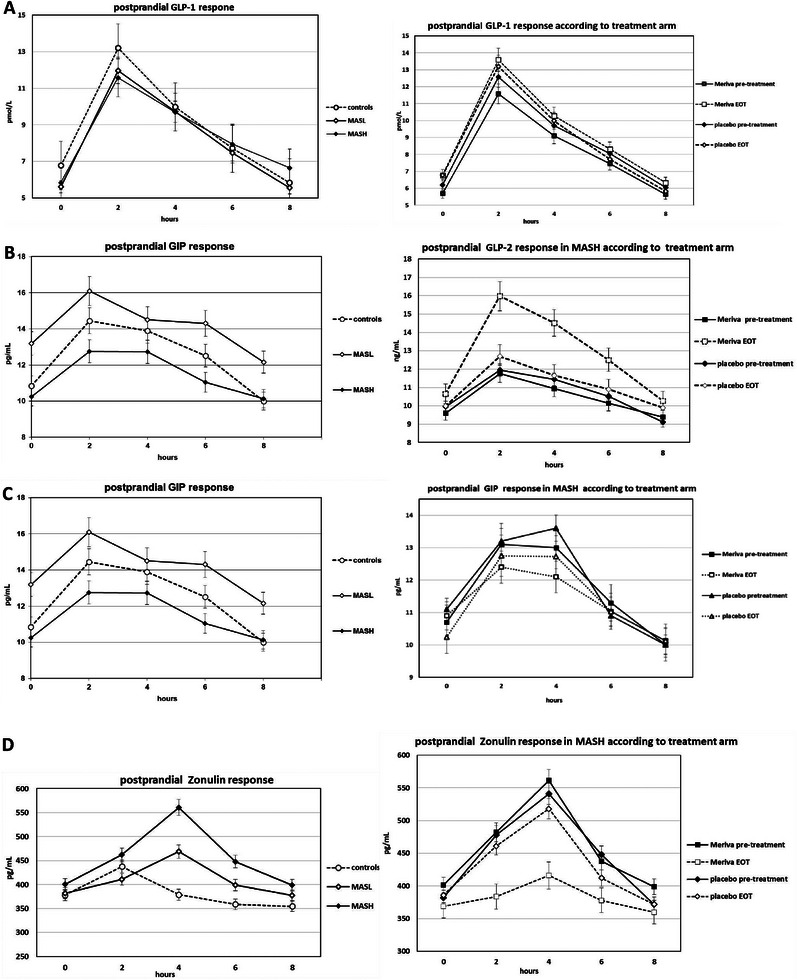
Oral fat tolerance test (OFTT): postprandial response in GLP‐1 (A), GLP‐2 (B), GIP (C) and Zonulin (C) in controls (*n* = 52), MASL (*n* = 52) and MASH (*n* = 52) patients in the cross‐sectional cohort (left panels) and in MASH patients enrolled in the Meriva trial (interventional cohort, *n* = 52, right panels). Data are expressed as mean (SEM).

Plasma CK‐18, MCP‐1, and lumican increased postprandially in MASH as compared with MASL and controls, indicating that fat ingestion can acutely trigger hepatocyte apoptosis, chemokine secretion, and fibrogenesis in MASH (Table S2).

In the interventional cohort, compared with baseline, MASH patients receiving Meriva showed an approximately threefold increase in IAUC GLP‐2, an improvement in intestinal barrier integrity (reduced IAUC Zonulin) and a reduction in endotoxemia, but they did not show any significant changes in postprandial GLP‐1, GIP, and other hormonal responses (Figure 1A–D, right panels).

In either treatment arms, MASH resolvers and fibrosis improvers showed a significant increase in postprandial GLP‐2 response as compared to patients who did not improve liver disease [mean(SEM) Δ IAUC GLP‐2 from baseline for MASH resolvers: +12.9(2.4) vs. −15.2(2.6) ng/mL x h, *p* = 0.0009); for fibrosis improvers: +13.4(2.9) vs. −15.7(3.1) ng/mL x h, *p* = 0.0001].

On multivariate analyses in the cross‐sectional cohort, IAUC GLP‐2 predicted the presence of MASLD (OR = 0.41, 95%CI: 0.22–0.63, *p* = 0.0007), MASH (OR = 0.45, 95%CI: 0.33–0.56, *p* = 0.0001), and significant (stage F ≥ 2) fibrosis (OR = 0.51, 95%CI: 0.32–0.73, *p* = 0.0008) independently of PNPLA3 C/G polymorphism, OGIS, IAUC LPS, IAUC zonulin, and IAUC NF‐kB activation in circulating MNCs. In the interventional cohort, Δ IAUC GLP‐2 predicted MASH resolution (OR = 2.21, 95%CI: 1.13–3.29, *p* = 0.002), a ≥1 stage fibrosis improvement (OR = 2.79, 95%CI:1.62–3.94, *p* = 0.002), and clinically significant (stage F ≥ 2) fibrosis regression (OR = 2.94, 95%CI:1.73–3.57, *p* = 0.001), independently of treatment allocation, change in IAUC LPS, in IAUC zonulin, and in IAUC NF‐kB activation in circulating MNCs.

In conclusion, this study disclosed a progressive GLP‐2 secretion impairment, gut barrier integrity disruption, and endotoxemia in response to fat as key mediators of lipotoxicity and contributors to human MASLD presence and progression.

These findings point to GLP‐2 secretion and intestinal barrier integrity normalization as a central therapeutic target in MASLD. As none of proposed therapeutic approaches showed evidence of gut protection, restoring a normal GLP‐2 response may offer synergistic benefits with weigh‐losing approaches centered on GLP‐1/GIP analogs and may represent a valuable therapeutic strategy in nonobese/lean MASLD patients, who represent 19%–40% of MASLD population, who are not candidate to substantial weight loss despite similar or higher liver‐related risk than obese MASLD patients [10].

## Author Contributions

G.M. and S.P. designed the trial, acquired and analyzed data, drafted the work, approved the final version, and agreed to be accountable for all aspects of the work. They are the guarantor of the article.

A.M., F.D.M., A.C., D.T., G.P., M.C., F.M., and R.G. gave substantial contributions to the study design and data acquisition, revised the work critically for important intellectual content, approved the final version to be published, and agreed to be accountable for all aspects of the work.

All authors have read and approved the final manuscript.

## Ethics Statement

The study was conducted in accordance with the ethical principles of the Declaration of Helsinki and approved by A.O.U San Luigi Gonzaga Hospital Ethics Committee (prot. no. 0008942) on May 25, 2018.

All participants gave written informed consent to participate in the study.

## Conflicts of Interest

Giovanna Petrangolini is an employee in R & D Indena S.p.A., but has no potential relevant financial or non‐financial interests to disclose. The other authors have no conflicts of interest to declare.

## Supporting information




**Supporting Table 1**: Demographic, clinical, biochemical and histological characteristics of controls and MASLD patients (n = 156).
**Supporting Table 2**: Postprandial lipid metabolism and inflammatory response parameters of MASLD patients (n = 104) and controls (n = 52) during the oral fat tolerance test (OFTT).

## Data Availability

Deidentified individual participant data will be made available with publication to anyone upon reasonable request to the corresponding author Dr Giovanni Musso (e‐mail: giovanni_musso@yahoo.it), beginning after publication and up for 5 years.
