# Supplementary material for: Dysfunctional Lipid‐Induced Secretion of Glucagon‐Like Peptide‐2(GLP‐2), but Not of Incretins Glucagon‐Like Peptide‐1(GLP‐1)/Glucose‐Dependent Insulinotropic Polypeptide(GIP), Promotes Metabolic Dysfunction–Associated Steatotic Liver Disease (MASLD) Onset and Progression Through Gut Barrier Disruption and Endotoxemia
Source: MedComm (2020). 2026 Apr 2;7(4):e70323. doi: 10.1002/mco2.70323 (PMC13052185; doi:10.1002/mco2.70323)
Supplement: Supplementary file 1 — Supporting Table 1: Demographic, clinical, biochemical and histological characteristics of controls and MASLD patients (n = 156). Supporting Table 2: Postprandial lipid metabolism and inflammatory response parameters of MASLD patients (n = 104) and controls (n = 52) during the oral fat tolerance test (OFTT). [file MCO2-7-e70323-s001.docx]

**1**

**SUPPLEMENTARY MATERIAL**

**Dysfunctional lipid-induced secretion of Glucagon-like peptide-2(GLP-2), but not of incretins Glucagon-like peptide-1(GLP-1)/glucose-dependent insulinotropic polypeptide(GIP), promotes Metabolic dysfunction-associated steatotic liver disease (MASLD)  onset and progression through gut barrier disruption and endotoxemia**

Text………………………………………………………………………….pg. 2

Tables………………………………………………………………………..pg. 17

Figures……………………………………………………………………….pg. 28

**METHODS**

**Diagnosis of MASLD.** Metabolic dysfunction-associated Steatotic Liver Disease (MASLD) was diagnosed in the presence of

- hepatic steatosis by imaging, confirmed by liver biopsy *plus*
- at least one cardiometabolic risk factor **(supplementary Table 1**) *and*
- no other discernible cause of liver disease (detailed below)^[[1]](#endnote-1)^

**Upper abdomen ultrasound and** **computerized sonographic hepato-renal ratio for quantitative assessment of steatosis and exclusion of MASLD in controls**

**MASLD exclusion in controls**. The presence of MASLD or any other liver disease in controls was excluded by the absence of **all** of these criteria:

- hepatic steatosis was quantified using the computerized sonographic liver/kidney ratio using a predefined standardized protocol (detailed below). Sonographic liver/kidney ratio to quantitate liver steatosis has been previously validated by our and other groups against magnetic resonance imaging and liver histology in diabetic and nondiabetic individuals^22,^^[[2]](#endnote-2)^ ^[[3]](#endnote-3),^ ^[[4]](#endnote-4),^^[[5]](#endnote-5),^^[[6]](#endnote-6),^^[[7]](#endnote-7),^^[[8]](#endnote-8),^^[[9]](#endnote-9)^, and a computerized sonographic liver-kidney ratio cut-off <1.49 was shown to rule out histological and MRI-assessed hepatic steatosis >5% with 100% sensitivity
- liver enzyme elevation (including serum ALT>30 IU/L in men and >20 IU/L in women)^[[10]](#endnote-10)^.
- significant alcohol consumption (>20 g/d in males and >10 g/d in females, assessed by a validated questionnaire) or positive viral hepatitis or serological markers for any other liver disease, abnormal hepato-biliary ultrasound.

All examinations were performed with a GE Vivid7 ultrasound machine (GE Healthcare, Horten, Norway) equipped with a GE 4C curved array transducer (GE H4904PC). US studies were performed by two experienced radiologists (GM and EP) who were unaware of the patient’s clinical details and laboratory findings. All the instrument settings, including “gain,” “depth,” and “time-gain compensation,” were preset and fixed for each measurement. For assessment of US H/R ratio, ultrasound images with both liver and right kidney clearly visualized were obtained in the sagittal liver/right kidney view in the lateral position. US hepatic echo-intensity attenuation rate was assessed in right intercostals view at anterior axilla line in the supine position. All images were reviewed by one of the two radiologists involved in scanning. Analysis of digitized ultrasound images was performed by using NIHimage software (ImageJ 1.41o, National Institutes of Health, Bethesda, MD).

US hepatic/renal echo-intensity ratio

In sagittal liver/right kidney view, a region of interest (ROI) of 1.5 × 1.5 cm (1,296 pixels) in the liver parenchyma was selected. This area is large enough to calculate an average histogram value and small enough to avoid the inclusion of vessels or bile ducts in the specimen. Several ROI parameters were displayed including circumference and area, total brightness level, mean brightness level, SD, most frequent brightness level, and a histogram. Of all these parameters, we used only the mean brightness level for each organ (liver and right kidney) in this study.

Technically, the echo intensity can be influenced by many factors, particularly by gain intensity.

To avoid confounding by factors that can modify the echo intensity and thus bias comparisons,

the mean brightness levels of both the liver and the right kidney cortex were obtained on the

same longitudinal sonographic plane. The ratio between the mean brightness level of the liver and the right kidney was calculated manually to determine the hepatorenal sonographic index.

In each case, the calculation of the hepatorenal sonographic index was repeated at least twice.

When the difference was less than 0.20, the average was calculated. If there was a greater discrepancy, a third measure was performed and the average of the two closest measurements was used.

The ROI had to be as uniform as possible, excluding blood vessels, bile ducts, and other focal hypo/hyperechogenicity. Another ROI of 0.5 × 0.5 cm (144 pixels) was identified in the right renal cortex with no large vessels, renal sinus or medulla. To avoid the interference of depth-depended echo-intensity attenuation and the borderline echo distorting effects, the boundary between liver and right kidney area should be placed near the center of the image, and the liver and right kidney ROIs were selected at the same depth of the ultrasound images. The gray scale mean value of the mixels within the two ROIs was used as measurement of echo intensity. Then we divided the average hepatic gray scale by the average renal cortex gray scale to calculate the US hepatic/renal ratio.

US hepatic echo-intensity attenuation rate. In right intercostals view at anterior axilla line, a tangent line of the sector ultrasound image was drawn and the ultrasound wave transmission line was determined, starting from the point of tangency and perpendicular to the tangent line. Two ROIs of 1.5 × 1.5 cm (1,296 pixels) were selected in liver homogeneous regions along the ultrasound transmission line near the liver anterior margin (depth 4–6 cm) and the liver posterior margin, respectively. The linear distance between the two ROIs was also measured.

The echo intensity of ultrasound wave was attenuated exponentially, shown as the following equation:

*A A a f d*

d = 0 ×e− ⋅ ⋅ (1)

Where *A*0 and *A*d are ultrasound echo intensity at the sound source and the liver parenchyma at a specific depth, respectively; *a* is the attenuation coefficient of the liver parenchyma; *f* is the frequency of the ultrasound detector; *d* is the depth of ROI. The ratio of the average echo intensity in the liver near-field ROI to liver far-field ROI was then calculated based on equation (1):

*A A a f f n*

n f

d d =e ⋅ ⋅( − ) (2)

Where *A*n and *A*f are average ultrasound echo intensity in the near-field ROI and the far-field ROI, respectively; *a* and *f* have been defined in equation (1); d*n* and d*f* are the depth of liver near-field and far-field ROIs^[[11]](#endnote-11)^ ^[[12]](#endnote-12),^^[[13]](#endnote-13),^^[[14]](#endnote-14),^^[[15]](#endnote-15),^^[[16]](#endnote-16),^^[[17]](#endnote-17),^^[[18]](#endnote-18)^.

**Liver histopathology in MASLD**

One liver histopathologist (RP) with specific expertise in MASLD, blinded to the clinical, laboratory and (for MASH patients enrolled in the trial) study treatment allocation, performed all the histopathological assessments at the central trial site (HUMANITAS Gradenigo) using an in-house designed form. The histological diagnosis of MASH was established using H&E staining and haematoxylin van Gieson stains of formalin-fixed paraffin-embedded liver tissue. The histological diagnosis of MASH requireds a combination of >5% macrovesicular steatosis, hepatocyte ballooning (± Mallory’s Hyaline) and lobular inflammation (mixed infiltrate, related to foci of ballooning)^[[19]](#endnote-19)^. The assessment of ballooning is subjective, and thus for ‘uncertain’ hepatocyte ballooning, a key component of the diagnosis of MASH, ubiquitin immunohistochemistry wias used to identify material compatible with Mallory’s hyaline.

To validate the quality of the biopsy specimen, the core specimen length was measured and the number of portal tracts was recorded.

The NAFLD Activity Score(NAS) was calculated based on the Kleiner classification^[[20]](#endnote-20)^ The NAS is scored of 8, with 8 representing the highest activity. The NAS is the sum of scores of the 3 components of the histological scoring system, namely steatosis (0=<5%, 1=5–33%, 2=>33–66%, 3=>66%), lobular inflammation (0=no foci, 1=<2 foci/×200, 2=2–4 foci/×200, 3=>4 foci) and hepatocyte ballooning (0=none, 1=few ballooned cells, 2=many cells/prominent ballooning). The Kleiner scoring system for NAFLD fibrosis (F0-F4) was used to evaluate the stage of fibrosis in each biopsy specimen. Portal tract changes (inflammation, interface hepatitis, ductular reaction), an intrinsic feature of NASH, were also recorded^[[21]](#endnote-21)^.

**Exclusion of competing causes of Steatotic Liver Disease (SLD).** Criteria for excluding competing causes of Steatotic Liver Disease e were as follows: a history of alcohol consumption >20 g/d (males) and >10 g/d (females) (assessed by a detailed inquiry of patients and relatives and a validated questionnaire filled in daily for one week by the patients), positive serum markers of viral hepatitis B-C; exposure to occupational hepatotoxins or drugs known to be steatogenic or hepatotoxic; positive autoimmune or celiac disease markers; abnormal serum α_1_-antitripsin, ceruloplasmin, trasferrin saturation, or TSH. Mutations in the hemochromatosis genes HFE and TRF2 were detected in patients and controls using multiplex amplification reaction (Nuclear Laser Medicine, Milan, Italy). Liver iron concentration (LIC) and hepatic iron index (HII) were assessed from 2 mg dry weight tissue by atomic absorption spectroscopy.

**Dietary record with food frequency questionnaire.** Subjects were instructed to fill in a 7‑day validated and reproducible dietary questionnaire during an individual training session with a nutritionist; a list of foods is designed, for each item different portion sizes are specified according to the EPIC study^[[22]](#endnote-22)^. The diet record was analyzed using the WinFood database (Medimatica, Teramo, Italy) according to the table of food consumption of the Italian National Institute of Nutrition and Food Composition Database for Epidemiological Study in Italy^[[23]](#endnote-23)^. Based on FFQ data, excessive alcohol use was defined as .30 g/d for male individuals and .20 g/d for female individuals.

The FFQ was completed at baseline by all participants and at end-of-treatment for MASH patients enrolled in the randomized controlled trial with curcumin.

**Physical activity record.**

Physical activity (PA) was measured with the accelerometer SenseWear Armband (SWA), which had to be worn continuously on the nondominant arm for at least 3 consecutive days (2 working days and a week-end day) at baseline for all participants and at EOT for patients enrolled in the RCT

The SenseWear Armband (SWA) is a relatively new accelerometer device that can be used to quantify and monitor physical activity and energy expenditure (EE), which has been validated against indirect calorimetry^[[24]](#endnote-24)^ and has been shown to have acceptable reliaiability and reproducibility. The SWA is designed to be worn on the upper arm over the triceps. Its internal sensors include an accelerometer, a thermal flow sensor, a galvanic sensor that records skin response, a skin temperature sensor, and an air temperature sensor. The accelerometer in the armband has two axes and uses a microelectromechanical sensor that measures movement.

Physical activity is measured relative to gravity (1 mg=9.81 mm/sec2) with an interval of 20 ms and categorized based on intensity, according to White et al.^[[25]](#endnote-25)^ , into time spent in sedentary behavior (,48 mg) and in light (48–154 mg, e.g., walking), moderate (154–389 mg, e.g., cycling), and vigorous activities (.389 mg e.g., running). The software created by the manufacturer calculates then EE using a patented algorithm that combines acceleration, heat flow, and other parameters: technical and statistical procedures have been described in detail earlier**^[[26]](#endnote-26),^ ^[[27]](#endnote-27),^ ^[[28]](#endnote-28)^**..

**Genetic analyses.** Participants were genotyped for the single nucleotide polymorphisms (SNPs) rs738409 C>G in patatin-like phospholipase-3 (PNPLA3), the Transmembrane 6 superfamily member 2 gene (TM6SF2) TM6SF2 rs58542926 C>T variant, and for apoE genotype, a key regulator of postprandial lipid metabolism which has been previously linked to the risk of MASLD,

We used the real-time allele discrimination method, using TaqMan Allelic Discrimination Assay (Applied Biosystems, Foster city, CA). The TaqMan genotyping reaction was run on an 7300HT Fast Real-Time PCR (Applied Biosystem)

**Oral fat tolerance test(OFTT): meal composition**

Participants were encouraged to avoid strenuous physical efforts and to follow their usual diet during the 24 h preceding the test. The fat load consisted of a mixture of dairy cream (35% fat) and egg yolk for a total energy content of 766 kcal. The total amount of fat was based on the subject's body surface area (78.3 g fat 55.6 % saturated fatty acids, 29.6 % monounsaturated fatty acids, 4.2 % polyunsaturated fatty acids, 0.5 g cholesterol per m^-2^). The fat load was consumed during a period of 5 min; subjects kept fasting on the test morning and strenuous activity was forbidden, since exercise can reduce postprandial lipemia. Blood samples were drawn every 2 hours for 8 hours and the following parameters were measured in fasting conditions and postprandially at each time of the test:

**Other laboratory assessments.**

Fasting blood samples will be analysed for full blood count, urea, creatinine and electrolytes, thyroid stimulating hormone (TSH), lipid profile (total cholesterol, high density lipoprotein, triglycerides), liver function tests, prothrombin time, international normalised ratio (INR), amylase, α-fetoprotein, C reactive protein, HbA1c,

Monocyte chemoattractant protein-1(MCP-1), and plasma glucose ( glucose was measured by the glucose oxidase method (Sentinel) with an intra-assay variation coefficient of 1.07 % and an inter-assay variation coefficient of 2.33%.) using standard laboratory methods (Roche Modular system, Roche Ltd, Lewes, UK). Urinary albumin excretion rate will measured using standard routine methods.

Serum Insulin (Mercodia, Uppsala, Sweden), nonesterified fatty acids (Zen-Bio, Research Triangle Park,North Carolina, USA)

**Oral fat load test: HDL-C determination**

Total HDL-Chol(HDL-C) was determined by enzymatic colorimetric assay after precipitation of LDL and VLDL fractions using heparin-MnCl2 solution and centrifugation at 4°C and it had an intra-assay variation coefficient of 2.5 % and an inter-assay variation coefficient of 4.1%.

**Oral fat load test: apolipoprotein determination**

**Apo B48 w**as measured with a sandwich-ELISA as the method. During incubation apo B48 in the sample reacts with anti-apo B48 antibody bound to microtitration well. After washing, which removes non-reactive plasma components, a biotinylated detection antibody specific for apo B48 and avidin-horseradish peroxidase conjugate is added to each micro plate well successively and incubated. After a second incubation and a simple washing step that removes unbound enzyme labeled antibody, the bound conjugate is detected by reaction with the substrate solution. The reaction is stopped by adding acid to give a colorimetric endpoint, then read spectrophotometrically (Elabscience, China).

The determination of **apolipoprotein A1** is based on the turbidimetric specific reaction which occurs between the anti- apolipoprotein A1 polyclonal antiserum and its corresponding antigen in oplimal pH conditions and in the presence of poly- ethylenglycole polymer (PEG) The turbidity of the immuno- complex is proportional to the concentration of the analites in the examined sample (Roche).

**Oral fat load test: enteroendocrine hormones, Inflammatory markers, hepatokine, adipokines**

Serum CRP levels were determined via a high sensitivity latex agglutination method on HITACHI 911 Analyser (Sentinel Ch., Milan). The kit had a minimum detection of less than 0.05 mg/L, and a measurable concentration range up to 160 mg/L. The intra-assay and inter-assay variation coefficients were, respectively, 0.8–1.3 and 1.0–1.5%.

Serum total **GLP-1** was measured by sandwich ELISA (DRG Instruments GmbH, Marburg, Germany). The kit has a sensitivity of 0.6 pmol/L in a 100‑μL sample size and a range of 2 to 48 pmol/L. The intra‑ and inter‑assay coefficients of variation were 3.7 % and 6.2%, respectively. Assay standards, controls and test samples are directly added to wells of a microplate that is coated with streptavidin. Subsequently, a mixture of biotinylated GLP-1 specific antibody and a horseradish peroxidase (HRP) conjugated GLP-1 specific antibody is added to each well. After the first incubation period, a “sandwich” immunocomplex of “Streptavidin – Biotin-Antibody – GLP-1(7-36)/(9-36) – HRP conjugated antibody” is formed and attached to the wall of the plate. The unbound HRP conjugated antibody is removed in a subsequent washing step. For the detection of this immunocomplex, each well is then incubated with a substrate solution in a timed reaction and then measured in a spectrophotometric microplate reader. The enzymatic activity of the immunocomplex bound to GLP-1 (7-36)/(9-36) on the wall of the microtiter well is directly proportional to the amount of Total GLP-1 in the sample.

**FGF-21**  was measured by sandwich ELISA, (BioVendor, Brno, Czech Republic) in serum samples. Samples and standards are incubated in microtitrate wells pre-coated with polyclonal anti-human FGF-21 antibody. After 60 min incubation and a washing, biotin-labelled polyclonal anti-human FGF-21 antibody is added and incubated with captured FGF-21 for 60 min. After another washing, the streptavidin-HRP conjugate is added. After 30 min incubation and the last washing step, the remaining conjugate is allowed to react with the substrate solution (TMB). The reaction is stopped by addition of acidic solution, and absorbance of the resulting yellow product is measured. The absorbance is proportional to the concentration of FGF-21. A standard curve is constructed by plotting absorbance values against concentrations of standards, and concentrations of unknown samples are determined using this standard curve.

**GLP-2** was measured by EIA (DRG Instruments GmbH, Marburg, Germany). The kit has a sensitivity of 0.4 ng/mL in a 25‑μL sample size and a range of 0.412 to 100 ng/mL. The intra‑ and inter‑assay coefficients of variation were 4 % and 14%, respectively. The EIA kit for determination of human GLP-2 in plasma and serum samples is based on a competitive enzyme immunoassay using combination of highly specific antibody to total human GLP-2 and biotin–avidin affinity system.

To the wells of the plate, coated with goat anti rabbit IgG, standard antigen or samples, biotinylated human GLP-2 and rabbit anti GLP-2 antibody are added for competitive immunoreaction. After incubation and plate washing, horse radish peroxidase (HRP) labelled-streptavidin (SA) is added to form HRP-labelled SA – biotinylated GLP-2 – antibody complex on the surface of the wells. Finally, HRP enzyme activity is determined by o-phenylenediamine dihydrochloride (OPD) and the concentration of human GLP-2 is calculated.

**LPS:** The Hycult Biotech Limulus Amebocyte Lysate assay is a sensitive and specific product available to detect and measure bacterial endotoxin. (Hycult biotech, Uden The Netherlands). The kit has a minimum detection of 0.04 EU/mL The intra-assay coefficients of variations (CVs) was, as average, 1.9%. The kit has a minimum detection of 0.04 EU/ml. Samples were diluted with endotoxin-free water then heated at 75 °C for 5 min in a warm plate to neutralize endotoxin inhibiting compounds. Manufacturer directions were followed.

Serum total **GIP** was measured by sandwich ELISA (Linco, St. Charles, Missouri, USA). The kit has a sensitivity of 8.2 pg/mL in a 20‑μL sample size and a range of 8.2 to 2000 pg/mL. The intra‑ and inter‑assay CVs were 3. % and 2.3%, respectively.

Serum **Zonulin** was measured by sandwich ELISA (AssayGenie, Dublin, Ireland). Diluted serum samples were assayed according to the manufacturer’s instructions. The kit has a sensitivity of 0.47 ng/mL in a 100 μL sample size and a range of 0.78 to 50 ng/mL. The intra  and inter assay coefficients of variation were between 4.85 - 5.69%, and 4.62 – 5.19 %, respectively. No significant cross-reactivity or interference between human zonulin and analogies were observed

Serum **LPS**  was measured with the Limulus Amoebocyte Lysate chromogenic end-point assay (Hycult Biotechnology, Uden, the Netherlands; inter- and intra-assay coefficients of variation, 4.5%; sensitivity, 1.0 EU/mL).

Serum **adiponectin** was measured by sandwich enzyme-linked immunosorbent assays (BioVendor, Brno, Czech Republic). The kit has a sensitivity of 470 ng/mL in a 100-μL sample size and a range of 5000 to 150000 ng/mL. The intra-and inter-assay coefficients of variations were 4.1% and 6.9%, respectively. All samples were diluted 1/300.

**Resistin** was measured by a biotin-labeled antibody- based sandwich enzyme immunoassay

(BioVendor Laboratory Medicine, Brno, Czech Republic). The intra-assay e inter-assay CVs were respectively 2.8–3.4% and 5.5– 6.8%.

**Redox balance.**

Plasma **total antioxidant status (TAS)** measurement was based on the reduction of Cu++ into Cu+ by the action of all present antioxidants. The amount of Cu+ is evaluating through measuring the complex formed by Cu+ and bathocuproine. This complex has a typical absorption at 490 nm. (ANTOXT Kit by Fujirebio Diagnostics AB Göteborg, Sweden)^.^

**Ox-LDLs w**ere measured with a solid phase two-site enzyme immunoassay. It is based on the direct sandwich technique in which two monoclonal antibodies re directed  against separate antigenic determinants on the oxidized apolipoprotein B molecule. During incubation oxidized LDL in the sample reacts with anti-oxidized LDL antibodies bound to microtitration well. After washing, which removes non-reactive plasma components, a peroxidase conjugated anti-human apolipoprotein B antibody recognizes the oxidized LDL bound to the solid phase. Alter a second incubation and a simple washing step that removes unbound enzyme labeled antibody, the bound conjugate is detected by reaction with 3,3', 5,5'-tetramethylbenzidine (TMB). The reaction is stopped by adding acid to give a colorimetric endpoint, then read spectrophotometrically (Mercodia, Uppsala, Sweden).

**Oral fat load test: Nuclear Factor(NF)-κB activation in mononuclear cells (MNCs)**

Blood samples were collected in tubes containing Na-EDTA as an anticoagulant; 3.5 mL of the anticoagulated blood sample was carefully layered over 3.5 mL of the PMNL isolation medium (Robbins Scientific Corp, Sunnyvale, CA). Samples were centrifuged at 450 x *g* in a swing outrotor for 30 min at 22 °C. At the end of centrifugation, 2 bands separate out at the top of the red blood cell pellet. The top band consists of monocuclear leukocytes(MNCs), whereas the bottom band consists of polymorphumuclear leukocytes(PMNs). The MNC and PMN bands were harvested with a Pasteur pipette, repeatedly washed with Hank’s balanced salt solution, and reconstituted to a concentration of 4 x 10^5^ cells/mL in Hank’s balanced salt solution. This method yields> 95% pure PMN and MNC suspensions(^[[29]](#endnote-29)^).

NF-kBp50/p65 transcription factor assay kit was purchased from Cayman Chemical (Ann Arbor MI, USA). The method detects specific transcription factor DNA binding activity in nuclear extracts and cell lysates. A specific double stranded DNA sequence containing the NF-kB response element is immobilized onto the bottom of wells of a 96 well-plate. NF-kB in nuclear or cytoplasmatic extract binds specifically to the NF-kB response element. NF-kBp50/65 is detected by addition of specific primary antibody directed against NF-kBp50/65. A secondary antibody conjugated to Horseradish peroxidase is added to provide a sensitive colorimetric readout at 450 nm.

**Oral fat load test: markers of hepatocyte apoptosis and active fibrogenesis.**

Circulating **cytokeratin-18 fragments** were measured with the M30-Apoptosense ELISA kit, a one step in vitro immunoassay for the quantitative determination of the apoptosis-associated CK18Asp396 neo-epitope in serum (PEVIVA AB, Bromma, Sweden), has a sensitivity of 25 U/L in a 25- L sample size and a range of 75 to 1000 U/L. The intra- and inter-assay CVs are less than 8%.

Serum **lumican** was measured by sandwich ELISA (RayBiotech, Norcross, Georgia, USA). The enzyme-linked immunosorbent assay has a sensitivity of 0.1 ng/mL in a 100‑μL sample size and a range of 0.1 to 25 ng/mL. The intra‑ and inter‑assay coefficients of variation were <10 % and <12%, respectively.

**Liver immunohistochemistry for NF-κB activation in the liver in MASLD**

The pro-inflammatory transcription factor **NF-κB**  is a master regulator at the crossroads of metabolic, oxidative, immune and inflammatory pathways. NF-κB activation in all liver cell subtypes and in peripheral mononuclear cells (MNCs) plays a central role in MASLD pathogenesis and progression^35^. We therefore assessed NF-κB activation in the liver by immunohistochemistry.

The ImmunoCruz® Staining System will be used in deparaffinized liver tissue according to the manufacturer’s instructions (Santa Cruz Biotechnology, Santa Cruz, CA). Briefly, slides will be soaked in 3% hydrogen peroxide for 5 min, washed, and incubated in serum blocking solution for 20 min. Specimens will be then incubated with primary antibodies for 2 h at 37◦ C. Tissue samples will be probed with mouse monoclonal antibodies reactive to NF-*κ*B p65 (Santa Cruz Biotechnology). After rinsing, specimens was incubated with biotinylated secondary antibody and a horseradish peroxidase-streptavidin complex, for 30 min each. Tissue samples were then colorized with DAB substrate, counterstained, mounted, and examined. NF-*κ*B immunoreactivity was expressed as the % of positive cells/high-power field (×400)^[[30]](#endnote-30)^ and was also semiquantitatively evaluated using a 4-point scoring system (0—no staining; 1—positive staining in *<*30% of cells/high-power field; 2— positive staining in 30–70% of cells/high-power field; 3—positive staining in *>*70% of cells/high-power field).

**Outcomes**

Outcomes evaluated were

1)in the cross-sectional cohort: the presence of MASLD, of MASH and of clinically significant (i.e. stage ≥F2) fibrosis.

2) in the interventional cohort, we explored the following histological outcomes, in line with current recommendations of regulatory agencies for development of MASH pharmacological agents^[[31]](#endnote-31),^^[[32]](#endnote-32)^:

- histological MASH resolution (defined by the NASH Clinical Research Network as no more than mild residual inflammatory cells [score of 0 or 1] and no hepatocyte ballooning [score of 0]) **and** no worsening of liver fibrosis (with worsening defined as an increase of at least one stage on the Kleiner fibrosis classification scale) after 72 weeks, in line with regulatory perspectives.
- a ≥1 stage improvement in fibrosis and no worsening of NASH (with worsening defined as an increase of ≥1 point in either the lobular inflammation score or the hepatocyte ballooning score according to the NASH Clinical Research Network criteria) after 72 weeks
- regression of clinically significant (i.e. stage≥2) liver fibrosis^[[33]](#endnote-33)^, defined by the proportion of patients with baseline fibrosis stage 2-4 who had fibrosis stage 0-1 at EOT, without worsening of NASH.

Additionally, we also explored the predictors of the following outcomes at baseline (cross-sectional cohort) and at end-of-treatment (EOT)(interventional cohort): NAS score, Fibrosis stage, serum CK-18 and lumican, Zonulin (as a marker of intestinal permeability), LPS, NF-kB activation in the liver and in circulating MNCs,

**Statistical analyses**

**Statistical Analysis.** Data are expressed as mean standard error of the mean (SEM). Differences across groups were analyzed by analysis of variance (ANOVA) and then by Bonferroni’s correction, when variables were normally distributed; otherwise, Kruskal-Wallis’ test, followed by Dunn’s post-hoc test, was used to compare nonparametric variables. Normality was evaluated by Shapiro-Wilk’s test. Fisher’s exact test or the chi-square test were used to compare categorical variables, as appropriate. Area under the curve (AUC) and incremental AUC test during the OFTT were computed by the trapezoid method.

Adjustments for multiplicity testing in the primary analysis was performed with the Benjamini-Hochberg procedure.

Analysis of dietary, anthropometric, and metabolic parameters was made using Pearson’s correlation test, after log-transformation of skewed variables.

Multivariate repeated-measures ANOVA was used to test the interaction between time and group during the oral fat load test. When a significant interaction was found between factors, differences across groups were analyzed by ANOVA followed by Bonferroni’s correction, if variables were normally distributed; otherwise, Kruskal-Wallis’ test was performed, followed by Dunn’s post-hoc test, to compare nonparametric variables. Differences were considered statistically significant at P < 0.05.

A logistic regression model was used to identify independent predictors for categorical outcome meausres and adjusted Odds ratios (ORs) were calculated using the Mantel-Haenszel test.

Predictors of continuous outcome variables were analyzed using linear univariable and multivariable regression analysis. We searched the best model fit among four predictive models (linear, exponential, logarithmic, binomial) using R-squared values.

In all multivariable models we used a combination of stepwise backward procedure and exclusion of highly collinear variables through model-dependent Variance Inflation Factor(VIF) cut-off values to select covariates^[[34]](#endnote-34)^,^[[35]](#endnote-35)^ .We first calculated the VIFs for each effect in the usual manner by fitting the full model with all effects. Then, we implemented stepwise routine to remove variables causing loss of precision in parameter estimates by starting with the variable having the largest VIF. The first model-dependent cutoff value thus set the limit by VIF(1)= 1/(1-R_1_^2^). where R_1_^2^ is the coefficient of determination calculated by regressing the response on just that independent variable having the largest VIF in the full model.

If the VIF for that variable was less than VIF(1), then the collinearity among the independent variables was considered small enough to be ignored and all of the variables were kept in the model.

If the VIF for that variable was greater than or equal to VIF(1), then the other independent variables in the model had a better explanatory power for this variable than this variable had for the response and the variable was removed from the model.

The model fitness was ultimately checked using the Hosmer and Lemeshow goodness of fit

test (Prob *>* chi2 = 0.8245)^[[36]](#endnote-36)^.

All evaluable patients were analyzed on an intention-to-treat basis, and **a**ll tests were performed at two-tails with significance set at a p value <0.05. Patients with missing data considered as non-responders.

All analyses were carried out with Easy R ver1.61, Saitama, Japan^[[37]](#endnote-37)^.

**Table S1.** Demographic, clinical, biochemical and histological characteristics of controls and MASLD patients (n=156).

|  | **Controls**  **(n=52)** | **MASLD (n=104)** | |  |
| --- | --- | --- | --- | --- |
|  |  | **MASL**  **(n=52)** | **MASH**  **(n=52)** | **P**  **MASL vs.**  **MASH** |
| **Age (years)** | 54 (12) | 54 (11) | 54 (10) | 0.912 |
| **Male** | 26 (50%) | 26 (50%) | 26 (50%) | 0.889 |
| **Caucasian white race**  **Hispanic race** | 51(100%)  1(2%) | 51(94%)  1(2%) | 52(100%)  0(0%) | 0.828  0.815 |
| **Weight status**  **Obesity***  **Overweight*** | 35(67%)  17 (33%) | 35(67%)  17 (33%) | 35(67%)  17 (33%) | 0.999  0.999 |
| **Abdominal obesity*** | 41 (79%) | 41 (79%) | 41 (79%) | 0.999 |
| **Dysglycemia***  **Prediabetes**  **Type 2 diabetes** | 11(21%)  25(48%) | 10(19%)  25(48%) | 13(25%)  25(48%) | 0.569  0.613 |
| **Hyperlipidaemia** | 12 (23%) | 13 (25%) | 12 (23%) | 0.878 |
| **Hypertension*** | 31 (60%) | 32 (62%) | 30(58%) | 0.896 |
| **MetSy* n (%)** | 32(61%) | 32(61%) | 32(61%) | 0.999 |
| **ApoE n(%)**  **2-3**  **3-3**  **3-4** | 10(20%)  30(60%)  10(20%) | 11(22%)  30(57%)  11(21%) | 10(20%)  29(55%)  13(25%) | 0.895  0.724  0.812 |
| **PNPLA3 C/G n(%)**  **C/C**  **C/G**  **G/G** | 30(58%)†  15(30%)  7(12%) | 15(28%)  23(44%)  15(28%) | 13(25%)  24(47%)  15(28%) | 0.478  0.791  0.482 |
| **TM6SF2 C/T n(%)**  **CC**  **CT**  **TT** | 44(85)  7(12)  1(3) | 41(79)  8(15)  3(6) | 37(72)  10(20)  4(8) | 0.792  0.684  0.318 |
| **Physical activity (PA)** |  |  |  |  |
| **Light PA (min/d)** | 169.9 (36.4) | 171.8 (31.5) | 184.4 (38.9) | 0.813 |
| **Moderate PA (min/d)** | 102.6 (23.2) | 98.6 (22.1) | 99.7 (15.9) | 0.849 |
| **Vigorous PA (min/d)** | 8.3 (8.2) | 8.1 (7.3) | 9.2 (7.2) | 0.728 |
| **Sedentary time (min/d)** | 782.7 (88.7) | 809.8 (80.4) | 838.4 (91.3) | 0.769 |
| **Dietary habits** |  |  |  |  |
| **Total calories (kcal/d)** | 2372 (169) | 2501 (140) | 2551 (131) | 0.759 |
| **Fat (% kcal/d)** | 33.0 (0.9) | 35.1 (0.8) | 34.2 (0.9) | 0.594 |
| **CHO (% kcal/d)** | 47.2 (1.8) | 49.2 (1.1) | 48.9(1.1) | 0.573 |
| **Fiber (g/d)** | 28.9 (2.1) | 26.5 (2.2) | 24.9 (1.9) | 0.692 |
| **Protein (% kcal/d)** | 18.5 (0.8) | 16.1 (0.0) | 15.1 (0.9) | 0.429 |
| **SFA (% total fat)** | 33.6(0.8) | 34.9 (0.4) | 34.4 (0.9) | 0.712 |
| **MUFA(% total fat)** | 46.6 (1.4) | 47.8 (1.3) | 48.2 (1.1) | 0.639 |
| **PUFA(% total fat)** | 13.2 (1.1) | 12.1 (0.8) | 14.1 (0.9) | 0.498 |
| **Body-mass index (kg/m²)** | 32.2(1.0) | 33.1(1.1) | 33.5 (1.2) | 0.716 |
| **Waist circumference (cm)** | 100.3 (4.7) | 106.1 (5.1) | 108.3 (4.2) | 0.239 |
| **Body fat (%)** | 24(2) | 25(3) | 27(2) | 0.317 |
| **Systolic blood pressure (mm Hg)** | 127 (14) | 130 (13) | 133 (12) | 0.594 |
| **Diastolic blood pressure (mm Hg)** | 76 (11) | 79 (11) | 78 (9) | 0.395 |
| **HbA1c (%)** | 6.01 (0.47) | 6.72 (0.51) | 6.71 (0.52) | 0.413 |
| **HbA1c (%) in T2DM** | 7.01 (0.79) | 7.01 (0.52) | 7.12 (0.56) | 0.693 |
| **HOMA-IR** | 4.5 (3.4) † | 6.6 (4.9) | 7.8 (4.1) | 0.529 |
| **Fasting plasma glucose (mg/dL)** | 106(24) | 118 (28) | 115 (26) | 0.315 |
| **Fasting insulin (uU/mL)** | 13.8(7,4) | 22.3 (8.4) | 26.8 (9.1) | 0.615 |
| **Alanine aminotransferase (U/L)** | 21 (8) | 71 (18) | 72 (22) | 0.213 |
| **Aspartate aminotransferase (U/L)** | 17 (21) | 51 (21) | 56 (24) | 0.393 |
| **γ-glutamyl transferase (U/L)** | 18 (11) | 92 (63) | 121 (71) | 0.692 |
| **Alkaline phosphatase (U/L)** | 27 (13) | 87 (25) | 86 (38) | 0.479 |
| **Total bilirubin (mg/dL)** | 0.51 (0.21) | 0.72 (0.24) | 0.70 (0.19) | 0.394 |
| **Albumin (g/dL)** | 3.9 (0.5) | 3.7 (0.6) | 3.8(0.7) | 0.529 |
| **Comuterized sonographic Hepato/Renal ratio** | 1.18 (0.11) ‡ | 2.34(0.31) | 2.69(0.40) | 0.293 |
| **NAFLD activity score (0–8)** | - | 2.8 (0·5) | 5.9 (0.8) | **0.002** |
| **Kleiner fibrosis stage (0–4)** | - | 0 (0) | 2.0 (0.6) | **0.0009** |
| **Kleiner fibrosis stages**  **F0**  **F1**  **F2**  **F3**  **F4** | - | 0(0%)  0 (0%)  0(0%)  0 (0%)  0(0%) | 1(4%)  13 (25%)  22(40%)  14 (25%)  2(5%) | 0.397  0.447  0.693  0.559  0.692 |
| **Clinically significant (stage ≥F2) fibrosis n (%)** | - | 0 (0%) | 38 (71%) | **0.010** |
| **Advanced fibrosis (stage 3-4) n (%)** | - | 0 (0%) | 16(31%) | 0.893 |
| Hepatic nuclear NF-kB  **(% positive cells)** | - | 23(2) | 49(3) | **0.0001** |

Data are presented as n(%) or mean ± SEM.

Abbreviations: Tg: triglyceride; C: cholesterol; FGF: Fibroblast Growth Factor; MASH: Metabolic dysfunction-associated steatohepatitis; MCP: monocyte chemoattractant protein; MNCs: mononuclear cells: NF-κB: nuclear factor-κB; HOMA-IR=homeostasis model assessment of insulin resistance; MetSy: metabolic syndrome; oxLDL: oxidized LDL; Tg: triglyceride

† p<0.05 vs. MASLD

‡ p<0.01 vs. MASLD

¶ p<0.001 vs. MASLD

§ p<0.001 vs MASH

* Defined by current guidelines Alberti KG, et al.; International Diabetes Federation Task Force on Epidemiology and Prevention; Hational Heart, Lung, and Blood Institute; American Heart Association; World Heart Federation; International Atherosclerosis Society; International Association for the Study of Obesity. Harmonizing the metabolic syndrome: a joint interim statement of the International Diabetes Federation Task Force on Epidemiology and Prevention; National Heart, Lung, and Blood Institute; American Heart Association; World Heart Federation; International Atherosclerosis Society; and International Association for the Study of Obesity. Circulation. 2009;120:1640-5.

**Abdominal** obesity was defined by a waist circumference

1. ≥94 cm in men and ≥80 cm in women (Europeans)
2. ≥90 cm in men and ≥80 cm in women (South Asians and Chinese)
3. ≥85 cm in men and ≥90 cm in women (Japanese)

**Hyperlipidaemia** was defined as recorded in the past medical history, as receiving lipid-lowering drugs (eg, statin, fibrate, ezetimibe), or both.

High Tg was definrd as plasma Tg≥150 mg/dl or lipid-lowering treatment

Low HDL-C was defined as ≤39 mg/dl in men and ≤50 mg/dl in women or lipid-lowering treatmen

High LDL-C was defined by LDL-C levels >160 mg/dL or on cholesterol-lowering therapy. LDL concentration was calculated using the Friedwald formula.

**Hypertension** was defined as BP≥130/85 mmHg or receiving an anti–hypertensive drug, or both.

* The presence at at least one of these criteria [overweight or obesity (overall or abdominal), dysglycemia, high Tg, low HDL-C, hypertension) *plus* hepatic steatosis *and* exclusion of competing causes of liver disease was used to define MASLD (see **supplementary Appendix**).

**Non-alcoholic fatty liver disease (NAFLD) activity score** is the algebraic sum of steatosis score, lobular inflammation score and hepatocyte ballooning score;

**Table S2.** Postprandial lipid metabolism and inflammatory response parameters of MASLD patients (n=104) and controls (n=52) during the oral fat tolerance test (OFTT).

|  | **Controls**  **(n=52)** | **MASL**  **(n=52)** | **MASH**  **(n=52)** | **P**  **MASL vs. MASH** |
| --- | --- | --- | --- | --- |
| **Lipids and apolipoproteins** |  |  |  |  |
| **Fasting Tg (mg/dL)** | 123 (29)† | 137(29) | 143 (22) | 0.413 |
| **IAUC Tg (mg/dL x hr)** | 159(27)‡ | 359(21) | 373(24) | 0.297 |
| **Fasting NEFA (mmol/L)** | 0.52(0.09)‡ | 0.56 (0.05) | 0.59 (0.05) | 0.213 |
| **IAUC NEFA (mmol/L x hr)** | 1.55(0.38)‡ | 2.72(0.43) | 3.18(0.48) | 0.396 |
| **Fasting LDL-C (mg/dL)** | 123 (6) | 134 (8) | 137 (8) | 0.428 |
| **IAUC LDL-C (mg/dL x hr)** | -8(7) | -11(8) | -10(8) | 0.316 |
| **Fasting HDL-C (mg/dL)** | 45 (5) † | 41 (4) | 38(4) | 0.518 |
| **IAUC HDL-C (mg/dL x hr)** | -20(4) | -19(4) | -22(4) | 0.397 |
| **Fasting total cholesterol (mg/dL)** | 185 (33) | 192 (43) | 196 (41) | 0.492 |
| **IAUC total cholesterol (mg/dL x hr)** | -29(11) | -31(11) | -27(9) | 0.621 |
| **Fasting apoA1 (mg/dL)** | 146(5) | 139(5) | 138(5) | 0.395 |
| **IAUC apoA1 (mg/dL)** | -37(12)† | -51(10) | -63(12) | 0.116 |
| **Fasting apoB48 (mg/dL)** | 28(11) | 49(12) | 58(12) | 0.294 |
| **IAUC apoB48 (mg/dL)** | 127(31) † | 157(32 | 168(29) | 0.273 |
| **Redox balance** |  |  |  |  |
| **Fasting oxLDL (IU/L)** | 32.7(14.2)‡ | 47.3 (17.5) | 50.8 (16.8) | 0.613 |
| **IAUC oxLDL(IU/L x hr)** | 13.1(14.9)‡ | 23.6(11.9) | 35.8(17.4) | **0.009** |
| **Fasting TAS (mmol/L)** | 0.49(0.07) | 0.42(0.05) | 0.37(0.05) | 0.481 |
| **IAUC TAS (mmol/L x hr)** | 0.56(0.12)‡ | -0.31(0.10) | -0.68(0.12) | **0.012** |
| **Glucose response** |  |  |  |  |
| **Fasting plasma glucose (mg/dL)** | 105 (23) | 118 (28) | 116 (24) | 0.397 |
| **IAUC plasma glucose** | -3(3) | -1(3) | 1(2) | 0.471 |
| **Fasting insulin (uU/mL)** | 13.8(7,4) | 22.3 (8.4) | 26.8 (7.1) | 0.379 |
| **IAUC insulin (uU/mL x hr)** | -3(2) | -2(3) | -1(2) | 0.394 |
| **Enteroendocrine hormones** |  |  |  |  |
| **Fasting GLP-1 (pmol/L)** | 6.7(2.2) | 5.8(1.9) | 5.9(2.0) | 0.713 |
| **IAUC GLP-1 (pmol/L x hr)** | 22.4(3.9) | 23.5.6(3.4) | 24.2(3.1) | 0.629 |
| **Fasting GLP-2 (ng/L)** | 10.6(1.5) | 11.0(1.8) | 9.8(1.7) | 0.491 |
| **IAUC GLP-2 (ng/L x hr)** | 30.4(3.4)‡ | 17.1(2.1)# | 7.4(2.4) | **0.001** |
| **Fasting GIP (pg/mL)** | 10.9(1.2) | 13.1(1.6) | 10.0(1.4) | 0.528 |
| **IAUC GIP (pg/mL x hr)** | 25.7(2.9) | 21.7(3.1) | 22.4(2.8) | 0.729 |
| **Intestinal barrier integrity** |  |  |  |  |
| **Fasting Zonulin (ng/mL)** | 373(32) | 382(31) | 401(39) | 0.579 |
| **IAUC Zonulin (ng/mL x hr)** | 403(32)‡ | 620(36)¶ | 907(41) | **0.001** |
| **Endotoxemia** |  |  |  |  |
| **Fasting LPS (IU/mL)** | 3.5(1.0) | 4.2(1.3) | 4.1(0.9) | 0.486 |
| **IAUC LPS (IU/mL x hr)** | 4.8(1.4)‡ | 10.2(2.9) | 22.4(3.1) | **0.0009** |
| **Hepatokines** |  |  |  |  |
| **Fasting FGF-21 (pg/mL)** | 256(29)‡ | 355(31) | 371(37) | 0.439 |
| **IAUC FGF-21 (pg/mL x hr)** | -342(37) | -376(19) | -361(13) | 0.491 |
| **Adipokines** |  |  |  |  |
| **Fasting adiponectin (ng(mL)** | 9093±831 | 10628±910 | 9052 ±804 | 0.714 |
| **IAUC adiponectin (ng/mL x hr)** | -4424±918‡ | -6947±710 | -8296±983 | 0.279 |
| **Fasting resistin (ng/mL)** | 3.9(1.2) | 3.7(1.4) | 4.1(1.1) | 0.829 |
| **IAUC resistin (ng/mL x hr)** | 1.1(1-3) | 1.9(1.3) | 2.1(1.1) | 0.419 |
| **Inflammatory activation** |  |  |  |  |
| **nuclear NF-κB in MNCs**  **(% total cellular NF-kB)** | 25(2) | 26(2) | 28(2) | 0.713 |
| **IAUC nuclear NF-κB in MNCs**  **(% total cellular NF-kB x hr)** | 67(18)‡ | 126(23) | 242(21) | **0.0008** |
| **Fasting MCP-1 (pg/mL)** | 175(28) | 181(30) | 187(28) | 0.312 |
| **IAUC MCP-1 (pg/mL x hr)** | 255(31)‡ | 377(44) | 571(39) | **0.0009** |
| **Hepatocyte apoptosis** |  |  |  |  |
| **Fasting CK-18 fragments M30 (iU/L)** | 153(18)‡ | 271(21) | 499(32) | **0.003** |
| **IAUC CK-18 (IU/l x hr)** | 124(13)‡ | 182(18) | 571(21) | **0.001** |
| **Active fibrogenesis** |  |  |  |  |
| **Fasting lumican (ng(mL)** | 127(11)‡ | 149(18) | 181(29) | 0.016 |
| **IAUC lumican (ng/mL x hr)** | 101(16)‡ | 181(23) | 376(29) | **0.009** |

Data are presented as n(%) or mean ± SEM.

Abbreviations: Tg: triglyceride; C: cholesterol; FGF: Fibroblast Growth Factor; MASH: Metabolic dysfunction-associated steatohepatitis; MCP: monocyte chemoattractant protein; MNCs: mononuclear cells: NF-κB: nuclear factor-κB; oxLDL: oxidized LDL; Tg: triglyceride

† p<0.05 vs. MASLD

‡ p<0.01 vs. MASLD

¶ p<0.001 vs. MASLD

§ p<0.001 vs MASH

1. European Association for the Study of the Liver; European Association for the Study of Diabetes; European Association for the Study of Obesity. EASL-EASD-EASO Clinical Practice Guidelines on the management of metabolic dysfunction-associated steatotic liver disease (MASLD): Executive Summary. Diabetologia. 2024 Jun 13. doi: 10.1007/s00125-024-06196-3. Epub ahead of print. [↑](#endnote-ref-1)
2. Webb M, Yeshua H, Zelber-Sagi S, et al. Diagnostic value of a computerized hepatorenal index for sonographic quantification of liver steatosis. AJR Am J Roentgenol. 2009;192:909-14. [↑](#endnote-ref-2)
3. Xia MF, Yan HM, He WY, et al. Standardized ultrasound hepatic/renal ratio and hepatic attenuation rate to quantify liver fat content: an improvement method. Obesity (Silver Spring). 2012 ;20:444-52. [↑](#endnote-ref-3)
4. Mancini M, Prinster A, Annuzzi G, et al. Sonographic hepatic-renal ratio as indicator of hepatic steatosis: comparison with (1)H magnetic resonance spectroscopy. Metabolism. 2009;58: 1724-30 [↑](#endnote-ref-4)
5. Zhang B, Ding F, Chen T, Xia LH, Qian J, Lv GY. Ultrasound hepatic/renal ratio and hepatic attenuation rate for quantifying liver fat content. World J Gastroenterol. 2014 Dec 21;20(47):17985-92 [↑](#endnote-ref-5)
6. Martín-Rodríguez JL, Arrebola JP, Jiménez-Moleón JJ, et al. Sonographic quantification of a hepato-renal index for the assessment of hepatic steatosis in comparison with 3T proton magnetic resonance spectroscopy. Eur J Gastroenterol Hepatol. 2014;26:88-94. [↑](#endnote-ref-6)
7. von Volkmann HL, Havre RF, Løberg EM,et al. Quantitative measurement of ultrasound attenuation and Hepato-Renal Index in Non-Alcoholic Fatty Liver Disease. Med Ultrason. 2013 ;15:16-22. [↑](#endnote-ref-7)
8. Kwon HJ, Kim KW, Jung JH, et al. Noninvasive quantitative estimation of hepatic steatosis by ultrasound: a comparison of the hepato-renal index and ultrasound attenuation index. Med Ultrason. 2016;18:431-437. [↑](#endnote-ref-8)
9. Borges VF, Diniz AL, Cotrim HP, Rocha HL, Andrade NB. Sonographic hepatorenal ratio: a noninvasive method to diagnose nonalcoholic steatosis. J Clin Ultrasound. 2013;41:18-25 [↑](#endnote-ref-9)
10. [Miyake T](http://www.ncbi.nlm.nih.gov.offcampus.dam.unito.it/pubmed?term=Miyake%20T%5BAuthor%5D&cauthor=true&cauthor_uid=22331365), [Kumagi T](http://www.ncbi.nlm.nih.gov.offcampus.dam.unito.it/pubmed?term=Kumagi%20T%5BAuthor%5D&cauthor=true&cauthor_uid=22331365), [Hirooka M](http://www.ncbi.nlm.nih.gov.offcampus.dam.unito.it/pubmed?term=Hirooka%20M%5BAuthor%5D&cauthor=true&cauthor_uid=22331365). Metabolic markers and ALT cutoff level for diagnosing nonalcoholic fatty liver disease: a community-based cross-sectional study. [J Gastroenterol.](/C:/Users/Utente/Downloads/gastroenterology.) 2012; 47: 696-703. [↑](#endnote-ref-10)
11. Webb M, Yeshua H, Zelber-Sagi S, et al. Diagnostic value of a computerized hepatorenal index for sonographic quantification of liver steatosis. AJR Am J Roentgenol. 2009;192:909-14. [↑](#endnote-ref-11)
12. Xia MF, Yan HM, He WY, et al. Standardized ultrasound hepatic/renal ratio and hepatic attenuation rate to quantify liver fat content: an improvement method. Obesity (Silver Spring). 2012 ;20:444-52. [↑](#endnote-ref-12)
13. Mancini M, Prinster A, Annuzzi G, et al. Sonographic hepatic-renal ratio as indicator of hepatic steatosis: comparison with (1)H magnetic resonance spectroscopy. Metabolism. 2009;58: 1724-30 [↑](#endnote-ref-13)
14. Zhang B, Ding F, Chen T, Xia LH, Qian J, Lv GY. Ultrasound hepatic/renal ratio and hepatic attenuation rate for quantifying liver fat content. World J Gastroenterol. 2014 Dec 21;20(47):17985-92 [↑](#endnote-ref-14)
15. Martín-Rodríguez JL, Arrebola JP, Jiménez-Moleón JJ, et al. Sonographic quantification of a hepato-renal index for the assessment of hepatic steatosis in comparison with 3T proton magnetic resonance spectroscopy. Eur J Gastroenterol Hepatol. 2014;26:88-94. [↑](#endnote-ref-15)
16. von Volkmann HL, Havre RF, Løberg EM,et al. Quantitative measurement of ultrasound attenuation and Hepato-Renal Index in Non-Alcoholic Fatty Liver Disease. Med Ultrason. 2013 ;15:16-22. [↑](#endnote-ref-16)
17. Kwon HJ, Kim KW, Jung JH, et al. Noninvasive quantitative estimation of hepatic steatosis by ultrasound: a comparison of the hepato-renal index and ultrasound attenuation index. Med Ultrason. 2016;18:431-437. [↑](#endnote-ref-17)
18. Borges VF, Diniz AL, Cotrim HP, Rocha HL, Andrade NB. Sonographic hepatorenal ratio: a noninvasive method to diagnose nonalcoholic steatosis. J Clin Ultrasound. 2013;41:18-25 [↑](#endnote-ref-18)
19. Sanyal AJ, Brunt EM, Kleiner DE, et al. Endpoints and clinical trial design for nonalcoholic steatohepatitis. Hepatology 2011;54:344–53. [↑](#endnote-ref-19)
20. Kleiner DE, Brunt EM, Van Natta M, et al. Design and validation of a histological scoring system for nonalcoholic fatty liver disease. Hepatology 2005;41:1313–21. [↑](#endnote-ref-20)
21. Brunt EM, Kleiner DE, Wilson LA, et al. Portal chronic inflammation in nonalcoholic fatty liver disease (NAFLD): a histologic marker of advanced NAFLD-clinicopathologic correlations from the nonalcoholic steatohepatitis clinical research network. Hepatology 2009;49:809–20. [↑](#endnote-ref-21)
22. Sacerdote C, Fiorini L, Dalmasso M. Alimentazione e rischi di cancro. Indagine su un campione di 10054 volontari residenti nell’area torinese. Torino: AGAT 2000. [↑](#endnote-ref-22)
23. Carnovale E, Marletta P. Food composition table. Istituto Nazionale della Nutrizione. Milano: EDRA 1997 . [↑](#endnote-ref-23)
24. Mielke GI, de Almeida Mendes M, Ekelund U, Rowlands AV, Reichert FF, Crochemore-Silva I. Absolute intensity thresholds for tri-axial wrist and waist accelerometer-measured movement behaviors in adults. Scand J Med Sci Sports. 2023 Sep;33(9):1752-1764 [↑](#endnote-ref-24)
25. White T, Westgate K, Wareham NJ, et al. Estimation of physical activity energy expenditure during free-living from wrist accelerometry in UK adults. PLoS One 2016;11(12):e0167472. [↑](#endnote-ref-25)
26. Santos-Lozano A, Hernández-Vicente A, Pérez-Isaac R, Santín-Medeiros F, Cristi-Montero C, Casajús JA, Garatachea N. Is the SenseWear Armband accurate enough to quantify and estimate energy expenditure in healthy adults? Ann Transl Med. 2017 Mar;5(5):97. doi: 10.21037/atm.2017.02.31 [↑](#endnote-ref-26)
27. Lopez GA, Brønd JC, Andersen LB, Dencker M, Arvidsson D. Validation of SenseWear Armband in children, adolescents, and adults. Scand J Med Sci Sports. 2018 Feb;28(2):487-495. [↑](#endnote-ref-27)
28. Bhammar DM, Sawyer BJ, Tucker WJ, Lee JM, Gaesser GA. Validity of SenseWear® Armband v5.2 and v2.2 for estimating energy expenditure. J Sports Sci. 2016 Oct;34(19):1830-8.  [↑](#endnote-ref-28)
29. Ghanim H, Abuaysheh S, Sia CL. Increase in plasma endotoxin concentrations and the expression of Toll-like receptors and suppressor of cytokine signaling-3 in mononuclear cells following a high-fat high-carbohydrate meal: implications for insulin resistance. Diabetes Care 2009;32:2281–2287 [↑](#endnote-ref-29)
30. Ribeiro PS, Cortez-Pinto H, Solá S, Castro RE, Ramalho RM, Baptista A, Moura MC, Camilo ME, Rodrigues CM. Hepatocyte apoptosis, expression of death receptors, and activation of NF-kappaB in the liver of nonalcoholic and alcoholic steatohepatitis patients. Am J Gastroenterol. 2004;99:1708-17. [↑](#endnote-ref-30)
31. Food and Drug Administration. Noncirrhotic nonalcoholic steatohepatitis with liver fibrosis: developing drugs for treatment. Guidance for industry: draft guidance. December 2018 (https://www.fda .gov/media/119044/download). [↑](#endnote-ref-31)
32. European Medicines Agency. Reflection paper on regulatory requirements for the development of medicinal products for chronic non-infectious liver diseases (PBC, PSC, NASH). November 2018 (https://www .ema.europa.eu/en/documents/scientific -guideline/reflection-paper-regulatory -requirements-development-medicinal -products-chronic-non-infectious-liver_en .pdf). [↑](#endnote-ref-32)
33. Rinella ME, Neuschwander-Tetri BA, Siddiqui MS, Abdelmalek MF, Caldwell S, Barb D, Kleiner DE, Loomba R. AASLD Practice Guidance on the clinical assessment and management of nonalcoholic fatty liver disease. Hepatology. 2023 May 1;77(5):1797-1835 [↑](#endnote-ref-33)
34. Trevor A. Craney & James G. Surles. Model-Dependent Variance Inflation Factor Cutoff Values, Quality Engineering 2002; 14:3: 391-403, [↑](#endnote-ref-34)
35. Midi H, Sarkar S, Rana S. Collinearity diagnostics of binary logistic regression model. Journal of

    Interdisciplinary Mathematics. 2013;253-267. [↑](#endnote-ref-35)
36. Fagerland, M. W., & Hosmer, D. W. A Generalized Hosmer–Lemeshow Goodness-of-Fit Test for Multinomial Logistic Regression Models. *The Stata Journal*, 2012;  *12*, 447-453. [↑](#endnote-ref-36)
37. Kanda Y. Investigation of the freely available easy-to-use software 'EZR' for medical statistics. Bone Marrow Transplant. 2013;48:452-8 [↑](#endnote-ref-37)
